# High-Throughput Transcriptomic and RNAi Analysis Identifies *AIM1*, *ERGIC1*, *TMED3* and *TPX2* as Potential Drug Targets in Prostate Cancer

**DOI:** 10.1371/journal.pone.0039801

**Published:** 2012-06-28

**Authors:** Paula Vainio, John-Patrick Mpindi, Pekka Kohonen, Vidal Fey, Tuomas Mirtti, Kalle A. Alanen, Merja Perälä, Olli Kallioniemi, Kristiina Iljin

**Affiliations:** 1 VTT Technical Research Centre of Finland, and Turku Centre for Biotechnology, University of Turku, Turku, Finland; 2 Institute for Molecular Medicine Finland (FIMM), University of Helsinki, Helsinki, Finland; 3 Haartman Institute, Department of Pathology, University of Helsinki, Helsinki, Finland; 4 HUSLAB, Department of Pathology, Helsinki University Central Hospital, Helsinki, Finland; 5 Department of Pathology, Turku University Hospital and University of Turku, Turku, Finland; University of Nebraska Medical Center, United States of America

## Abstract

Prostate cancer is a heterogeneous group of diseases and there is a need for more efficient and targeted methods of treatment. In this study, the potential of gene expression data and RNA interference technique were combined to advance future personalized prostate cancer therapeutics. To distinguish the most promising *in vivo* prevalidated prostate cancer drug targets, a bioinformatic analysis was carried out using genome-wide gene expression data from 9873 human tissue samples. In total, 295 genes were selected for further functional studies in cultured prostate cancer cells due to their high mRNA expression in prostate, prostate cancer or in metastatic prostate cancer samples. Second, RNAi based cell viability assay was performed in VCaP and LNCaP prostate cancer cells. Based on the siRNA results, gene expression patterns in human tissues and novelty, endoplasmic reticulum function associated targets *AIM1*, *ERGIC1* and *TMED3*, as well as mitosis regulating *TPX2* were selected for further validation. *AIM1*, *ERGIC1*, and *TPX2* were shown to be highly expressed especially in prostate cancer tissues, and high mRNA expression of *ERGIC1* and *TMED3* associated with *AR* and *ERG* oncogene expression. *ERGIC1* silencing specifically regulated the proliferation of *ERG* oncogene positive prostate cancer cells and inhibited ERG mRNA expression in these cells, indicating that it is a potent drug target in ERG positive subgroup of prostate cancers. *TPX2* expression associated with PSA failure and *TPX2* silencing reduced PSA expression, indicating that TPX2 regulates androgen receptor mediated signaling. In conclusion, the combinatorial usage of microarray and RNAi techniques yielded in a large number of potential novel biomarkers and therapeutic targets, for future development of targeted and personalized approaches for prostate cancer management.

## Introduction

Prostate cancer is the most commonly diagnosed malignancy and the second most common cause of cancer mortality in the Western male population [1]. However, prostate cancers form a heterogeneous group of diseases and some men are still diagnosed with high-grade disease and ultimately fail treatment [1], [2]. Despite the phenotypic and molecular heterogeneity of the disease there is a lack of robust and specific prognostic biomarkers to distinguish between indolent and aggressive cancers at early phases of the disease. Furthermore, due to the lack of efficient prognostic and therapeutic biomarkers, as well as targeted therapeutics, the clinical management is still far from personalized.

Besides regulating the development and maintenance of the prostate, androgens support the development and growth of most primary prostate cancers, and androgen receptor (AR) plays the role of an oncogene in prostate cancer [3]–[7]. Accordingly, androgen ablation is currently the treatment of choice for advanced prostate cancer. However, although androgen blockage initially results in a good treatment response, it is almost never curative [2]. Androgen-independent cancer cells typically start to appear during therapy, eventually leading to recurrent, hormone-refractory disease [8], [9]. In addition to prevailing alterations in AR expression and function, approximately half of prostate cancer samples harbor an oncogenic gene fusion combining androgen-regulated transmembrane protease serine 2 (*TMPRSS2*) with oncogenic ETS transcription factors [10]. Most frequently, the fusion partner is *ERG* (v-ets erythroblastosis virus E26 oncogene homolog, avian), followed by *ETV1* (ets variant 1), *ETV4*, and *ETV5* [11]–[13]. ERG mRNA is not expressed in healthy prostate tissues, but as a result of the *TMPRSS2-ERG* gene fusion early in carcinogenesis, a significant increase in ERG transcript levels can be detected in prostate cancers. ETS gene fusions promote multiple signaling pathways associated with cancer formation and progression, and ectopic *ERG* oncogene expression has been associated with a specific molecular signature in prostate cancer [14]–[19]. Although *ERG* activation mediated oncogenic processes may be bypassed in advanced prostate cancer, hormone-regulated expression of *ERG* has been described to persist also in castration resistant prostate cancer, supporting the importance of this rearrangement also in advanced disease [15], [20], [21]. Taken together, ETS fusions are key molecular alterations driving the development and progression of a distinct class of prostate cancers, and could therefore benefit from targeted therapy.

In recent years advanced molecular genetic techniques combined with development of novel bioinformatic analysis tools have offered efficient ways to examine tumor gene expression profiles, which facilitates biomarker discovery, as well as identification of potential novel drug targets. Gene expression profiling enables improved diagnosis and staging of the disease, provides information on treatment responses and leads to reduced side effects [22], [23]. RNA interference (RNAi) technique enables the exploration of the functional effect of individual genes on cancer cell characteristics, such as growth and survival, further advancing the development of targeted and personalized therapeutics [24]–[26]. In this study, the potential of these techniques was combined by pre-selecting the genes for RNAi functional assays using gene expression data. To identify potential vulnerabilities present in prostate cancers, a bioinformatic mRNA expression analysis was first carried out based on 9873 human tissue samples, including 349 prostate cancer and 147 non-malignant prostate samples, to distinguish prostate and prostate cancer tissue specific genes. Second, a RNAi high-throughput (HT) functional profiling of the selected *in vivo* prevalidated possible drug targets was performed in VCaP and LNCaP prostate cancer cell lines in order to identify genes and pathways essential for prostate cancer cell proliferation and survival. The results highlighted the potential of targeting endoplasmic reticulum (ER), oxidation, actin cytoskeleton and mitosis in prostate cancer management, and further validation identified *AIM1* (absent in melanoma 1), *ERGIC1* (endoplasmic reticulum-Golgi intermediate compartment protein 1), *TMED3* (transmembrane emp24 protein transport domain containing 3) and *TPX2* (targeting protein for Xklp2) as potential novel drug targets in prostate cancer.

## Methods

### *In Silico* Data Mining

The GeneSapiens database [27] was applied to bioinformatically explore the gene expression levels across 9783 human tissue samples. Briefly, GeneSapiens (http://www.genesapiens.org/) is a collection of 9873 Affymetrix microarray experiments. All samples are reannotated and normalized with a custom algorithm. The data are collected from various publicly available sources, including Gene Expression Omnibus and Array-Express and covers 175 different tissue types. Mean expression of each gene was determined in prostate cancer (n = 349), healthy prostate (n = 147), and all normal tissue samples (n* = *1476). The data from prostate cancer samples available in the GeneSapiens database were utilized also in the *in silico* coexpression analyses. The functional gene ontology annotations were analyzed for the co-expressed genes (R >0.5 and P<0.001) using DAVID functional annotation tool [28] and Ingenuity Pathway Analysis (IPA) Software (Ingenuity Systems Inc., Redwood City, CA, USA).

### Cell Culture

VCaP prostate cancer cells were received from Kenneth Pienta (University of Michigan, MI) or purchased from American Type Culture Collection (LGC Promochem AB, Borås, Sweden) and grown in RPMI-1640 medium (Invitrogen, Carlsbad, CA). LNCaP cells were received from Dr. Marco Cecchini (University of Bern, Switzerland) and maintained in T-Medium (Invitrogen, Carlsbad, CA). PC-3, DU145 and MDA-PCa-2b cells were purchased from American Type Culture Collection (LGC Promochem AB), and 22Rv1 cells from Deutsche Sammlung von Microorganismen und Zellkulturen GmbH (DSMZ, Braunschweig, Germany). The non-malignant EP156T prostate epithelial cells were received from Dr. Varda Rotter (Weizmann Institute of Science, Rehovot, Israel) and RWPE-1 cells purchased from American Type Culture Collection (LGC Promochem AB). Primary prostate epithelial cells (PrEc) were purchased from Lonza (Lonza Group Ltd, Basel, Switzerland). Androgen-independent LNCaPs and their parental counterparts were received from Dr. Zoran Culig (Innsbruck Medical University, Austria) and were grown in RPMI-1640 (Invitrogen) containing charcoal stripped or normal fetal bovine serum, respectively. Synthetic androgen R1881 was purchased from PerkinElmer.

### Gene Knock-down Using RNA Interference

Before screening, cell number was titrated for both VCaP and LNCaP cells separately to ensure that cell proliferation remained in a linear-exponential phase throughout the experiment. For the RNAi studies, four siRNAs per gene (HP GenomeWide, Qiagen) were plated onto 384-well plates (Greiner Bio-One, Frickenhausen, Germany), followed by addition of the transfection agent (siLentFect lipid reagent; Bio-Rad Laboratories, Hercules, CA) in Opti-MEM medium (Invitrogen) and an appropriate quantity of cells (1500–2000 per well), using automated liquid handling robot (Hamilton) and liquid dispenser (ThermoFisher). The final siRNA concentration was 13 nM. AllStars negative control (scrambled siRNA, Qiagen) and lipid only were used as negative controls, siRNAs against *KIF11* (kinesin family member 11; SI02653770) and *PLK1* (polo-like kinase 1; SI02223844) were used as positive controls. For the validation experiments cells were transfected with two siRNAs per gene (*AIM1*: SI03126704, SI03212846; *ERGIC1*: SI03164763, SI04302872; *TMED3*: SI00746711, SI00746718; *TPX2*: SI00097188, SI00097195) as described above in the appropriate plates.

### Cell Viability and Apoptosis Assay

CellTitre-Blue (CTB) and CellTiter-Glo (CTG) cell viability assays (Promega), and ApoONE apoptosis (induction of caspase -3 and 7 activities) assay (Promega) were performed according to the manufacturer’s instructions in response to 48 h or 72 h siRNA treatment. The results were scanned with EnVision Multilabel platereader (PerkinElmer/Wallac).

### Normalization and Statistical Analysis of siRNA Screen Results

The raw results obtained from cell viability and apoptosis assays were normalized using *B*-score [29], and siRNAs reducing cell viability by -2 SD from the median of the controls (corresponding to P<0.05) in at least two of the screens or inducing apoptosis by 3 SD (corresponding to P<0.01) were considered antiproliferative or pro-apoptotic hit siRNAs.

### Clinical Prostate Tissue Samples

The 33 primary prostate tumor samples (19 ERG oncogene positive and 14 ERG negative) and 3 non-malignant prostate samples utilized in this study have been described previously [30].

### Quantitative Reverse Transcriptase PCR

The validation of mRNA expression levels was performed using TaqMan quantitative reverse transcriptase PCR (qRT-PCR) analysis (Finnish DNA Microarray Centre, Centre for Biotechnology, University of Turku). RNA samples extracted with RNeasy Mini Kit (Qiagen) were reversely transcribed to cDNA (High Capacity cDNA Reverse Transcription Kit, Applied Biosystems) and PCR reaction samples were analyzed in 96-well or 384-well format. Quantitative RT-PCR was performed using ABI Prism 7900 (Applied Biosystems) and quantitation was carried out using the _ΔΔ_CT method with RQ manager 1.2 software (Applied Biosystems). Three replicate samples were studied for detection of target mRNA expression and β-actin was used as an endogenous control. The primers and probes were designed and selected with the help of Universal ProbeLibrary Assay Design Center (Roche Diagnostics) (Supporting Table S1).

### Western Blot Analysis

Whole-cell lysates were prepared using lysis buffer (62.5 mM Tris, 1% SDS, 5%, β-mercaptoethanol 10% glycerol, bromophenol blue). Antibodies used included anti-AR (1∶1,000, NeoMarkers, Thermo Fisher Scientific Inc., Fremont, CA), anti-PSA (1∶1,000, A0562, DakoCytomation, Glostrup, Denmark), as well as secondary Alexa Fluor (1∶4,000, Molecular Probes, Invitrogen) antibodies. β-actin (1∶5,000, antibody from Sigma) was used as a loading control. The signal was detected using Odyssey Infrared Imaging System (LI-COR Biosciences, Lincoln, NE) according to the manufacturer’s instructions.

### Statistical Analysis

The results are presented as the mean ± SD. Statistical analyses were performed using Student’s t-test (*, P<0.05; **, P<0.01; ***, P<0.001) and Pearson correlation coefficient.

## Results

### High-throughput Screening Results Highlight the Role of Endoplasmic Reticulum and Mitosis Related Genes in Regulating Prostate Cancer Cell Growth and Survival

**Figure 1:**
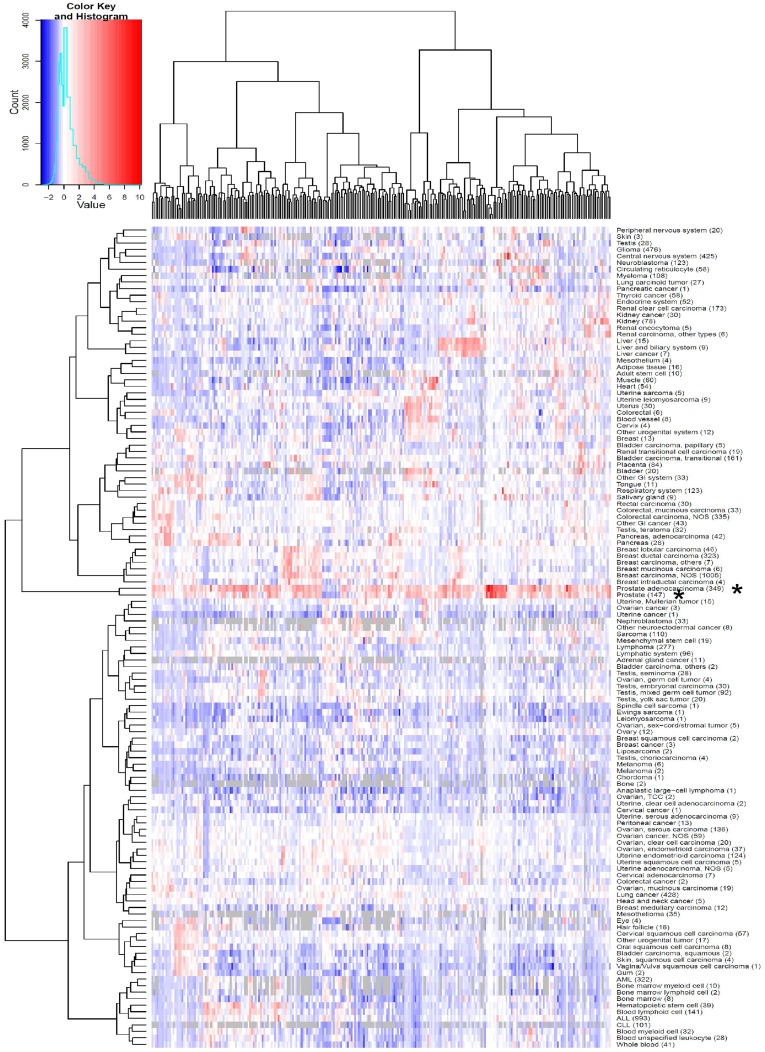
RNAi library target gene expression *in silico*. A heatmap presentation of the mean gene expression levels of the 295 genes (x-axis) selected for further RNAi exploration in all of the tissues (healthy and malignant) present in GeneSapiens database (y-axis). The position of prostate cancer (upper asterisk) and healthy prostate (lower asterisk) have been indicated. The colour illustrates the level of expression in different tissues, and grey missing values. The heatmap is drawn based on unsupervised hierarchical clustering.

To select *in vivo* prevalidated potential drug targets and biomarkers for further studies in cultured prostate cancer cells the gene expression data available in GeneSapiens database was utilized. In total, 295 prostate and/or prostate cancer specific genes were selected based on high mRNA expression in prostate, prostate cancer or in metastatic prostate cancer tissue samples, and an siRNA library was constructed for functional studies (Figure 1). For the RNAi studies 4 siRNAs per gene were purchased and plate based HT siRNA screens were performed with VCaP and LNCaP prostate cancer cell lines. VCaP is a model for TMPRSS2-ERG positive prostate cancer, expressing wild type AR, whereas LNCaPs harbour a mutant *AR* (T877A) with extended ligand specificity. To identify therapeutically relevant genes and pathways in prostate carcinogenesis, changes in cell viability and induction of apoptosis (caspase -3 and 7 activation) were studied as the end-points (Supporting Table S2).

**Figure 2:**
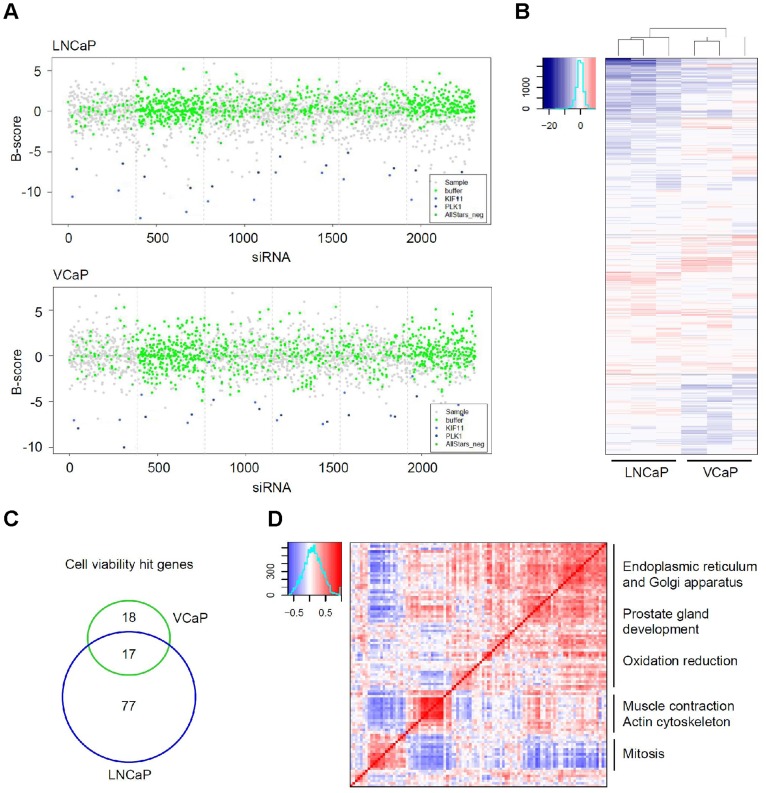
HT RNAi screening results. **A.** Overview of the normalized LNCaP and VCaP cell viability screen results (B-score). The results from the positive control siRNAs (KIF11 and PLK1) are indicated in blue, negative control wells (AllStars negative scrambled siRNA and buffer only) in green, and target gene siRNAs in gray. **B.** A heatmap presentation of the cell viability screen results (B-score). The assay was repeated three times in both LNCaP and VCaP prostate cancer cell lines. Blue colour indicated decreased cell viability, red increased cell viability. The heatmap is drawn based on unsupervised hierarchical clustering. **C.** The overlap between the RNAi screen hit genes (decreased cell viability in response to silencing) in LNCaP and VCaP cell lines. **D.** *In silico* co-expression analysis of cell viability hit genes in prostate cancer samples. The genes are organized in the same order in both y- and x-axis, and the correlations (R) between the genes are indicated with colours. Red indicates positive correlation, blue negative correlation.

The cell viability siRNA screen was performed in three replicates and the apoptosis assay once in both cell lines. The positive control siRNAs targeting known key regulators of the mitotic progression as well as prostate cancer cell proliferation, KIF11 and PLK1 [31], [32], were able to significantly decrease cell viability (Figure 2A) confirming thus transfection efficiency. The replicate cell viability screens positively correlated (0,67< R <0,78 in LNCaP and 0,36< R <0,66 in VCaP) in both cell lines supporting the functionality of the primary screens (Figure 2B and Supporting Table S2).

The siRNA screens resulted in 94 potential proliferation promoting (hits in at least two of the cell viability screens) and 97 anti-apoptotic genes in LNCaP cells. Out of the 94 reproduced cell viability hit genes 45 (47.9%) were also anti-apoptotic. In VCaP cells the final hit rate was 35 reproduced proliferation promoting and 34 anti-apoptotic hit genes, 9 (25.7%) of which promoted cell viability and protected from apoptosis. Silencing of 17 genes resulted in an anti-proliferative response in both LNCaP and VCaP cells. (Figure 2B-C and Supporting Table S2).

The *in silico* co-expression analysis of proliferation hit genes (n = 112) suggested three major prostate cancer sub groups with different mechanisms for cell growth regulation. The largest set of genes had a role in ER and Golgi apparatus, prostate gland development, as well as in oxidation reduction. The other subgroups of prostate cancer viability regulating genes were involved in actin cytoskeleton and mitosis (Figure 2D).

### Novel Putative Prostate Cancer Drug Targets *AIM1*, *ERGIC1*, *TMED3*, and *TPX2* were Selected for Further Validation

The RNAi screens confirmed the role of multiple previously published prostate cancer drug targets as growth and apoptosis regulating genes in cultured prostate cancer cells. Among others, these genes included *CLDN3*, *CYP4F8*, *EPHX2*, *FAAH*, *FOXA1*, *MTDH*, *ODC1*, *PLA2G2A*, *PLA2G7*, *SIM2* and *UBE2C* [30], [33]–[41] (Supporting Table S2).

Four novel candidate drug targets, *AIM1*, *ERGIC1*, *TMED3*, and *TPX2*, were selected for further studies based on the high expression in prostate cancer compared to normal prostate and all other normal tissues included in GeneSapiens database (Supporting Figure S1), as well as their novelty as regulators of prostate cancer cell proliferation and apoptosis. *AIM1*, *TMED3* and *TPX2* were among the 17 genes, the silencing of which induced antiproliferative effects in both VCaP and LNCaP cells as well as apoptosis in at least one of the cell lines. Silencing of *ERGIC1* induced antiproliferative effect specifically in the ERG oncogene positive VCaP cells (Supporting Table S2). *AIM1*, *ERGIC1* and *TMED3* were co-expressed in the set of genes functionally annotated to ER and Golgi apparatus and redox reactions, whereas *TPX2* was expressed among the genes involved in mitosis (Figure 2D).

AIM1 protein is a member of the βγ-crystalline superfamily. Unlike other β- and γ-crystallines, known to be specifically expressed in elongating lens fiber cells that are undergoing large changes in cytoskeletal architecture and composition, AIM1 has a non-lens role. However, AIM1 protein sequence has a weak similarity with filament or actin-binding proteins, indicating a possible role in the management of cell morphology and shape [42]. *AIM1* gene localizes in 6 q21, within the putative tumor suppressor region for human melanoma, and AIM1 expression has been shown to be altered in association with tumor suppression in a human melanoma model [43]. However, recent studies indicated that *AIM1* is not the main tumor suppressor gene in del6q21 in natural killer cell malignancies [44], [45]. Supporting the possible role of *AIM1* as a tumor suppressor, AIM1 methylation has been associated with nasopharyngeal carcinoma and primary tumor invasion of bladder cancer [46], [47]. On the other hand, AIM1 expression has been shown to be high in TRAIL resistant cancer cell lines [48].

ERGIC1 is a cycling membrane protein contributing to the membrane traffic and selective transport of cargo between the ER, the intermediate compartment, and the Golgi apparatus [49], whereas TMED3 is a constituent of the coated vesicles that are involved in the transportation of cargo molecules from the ER to the Golgi complex and function as receptors for specific secretory cargo [50]. Although the exact role of ERGIC1 and TMED3 in cancer remains to be elucidated, the dysfunction of proteostasis and ER is known to induce a stress response (unfolded protein response) leading to apoptosis in cancer cells [51], [52].

TPX2 is exclusively expressed in proliferating cells from the transition G1/S until the end of cytokinesis. Mitosis is a major biological process deregulated in cancer and the main biological process targeted by cytotoxic drugs. Interestingly, TPX2 is known to be highly expressed in various cancer tissues, and it has been suggested as a biomarker for poor prognosis [53]–[55]. As an important regulator of cell cycle and a binding partner for Aurora A kinase, TPX2 has been suggested also as a potential drug target in multiple malignancies [56]–[58]. However, TPX2 has not been studied in prostate cancer previously. It has been suggested that TPX2 targeted therapeutics could be more efficient than the use of Aurora A kinase inhibitors due to the unspecific nature of conventional kinase inhibitors [58]. Furthermore, combining TPX2 and Aurora A kinase targeted therapeutics could inhibit the development drug resistance [59], [60].

### Validation of *AIM1*, *ERGIC1*, *TMED3*, and *TPX2* Expression and siRNA Induced Target Gene Silencing in Cultured Prostate Cells

**Figure 3:**
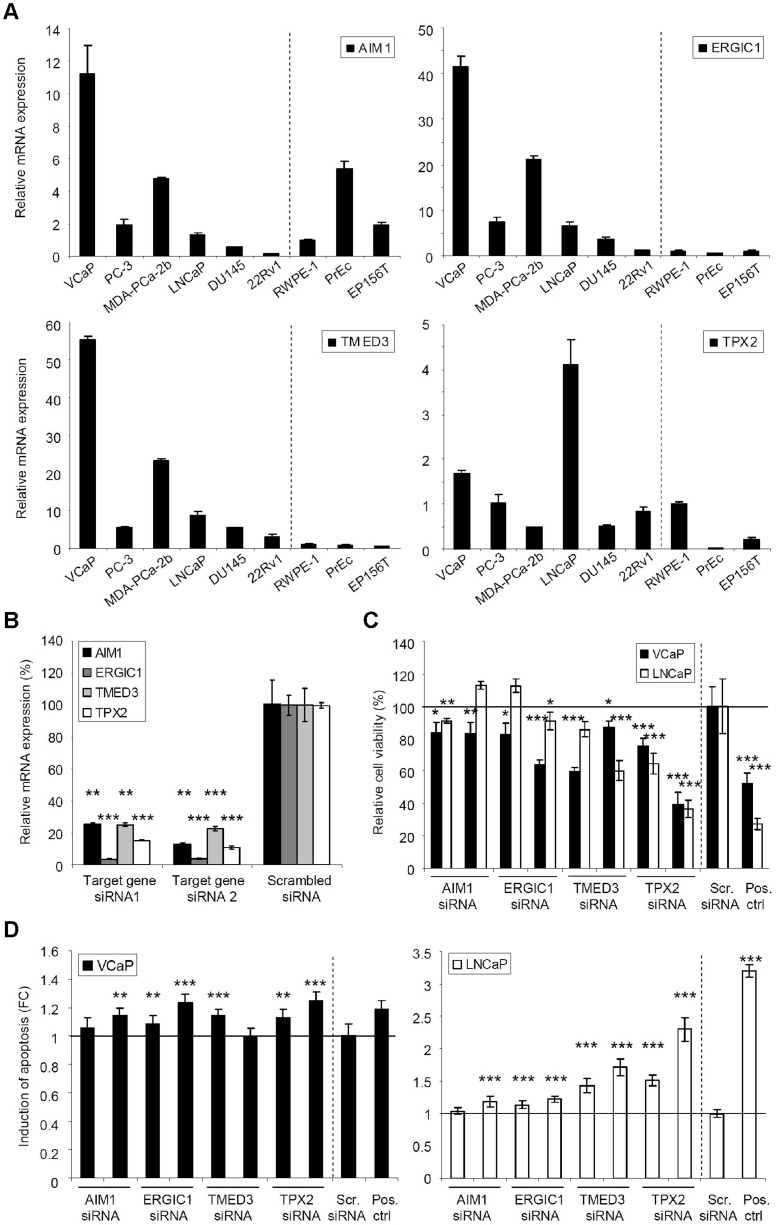
Validation of *AIM1*, *ERGIC1*, *TMED3* and *TPX2* expression and silencing in prostate cell lines. **A.** The mRNA expression of target genes in 6 prostate cancer (VCaP, PC-3, MDA-PCa-2b, LNCaP, DU145 and 22Rv1) and 3 non-malignant (RWPE-1, PrEc, EP156T) prostate cell lines. For each gene the relative mRNA expression in RWPE-1 cell line was set to 1. **B.** Validation of target gene silencing. The mRNA level of each gene in control sample has been set as 100%. **C.** The effect of target gene silencing on VCaP and LNCaP cell viability at 72 h timepoint. **D.** The effect of target gene silencing on induction of apoptosis in VCaP and LNCaP cells at 72 h timepoint. The results have been compared to scrambled siRNA induced changes and the significance of the anti-proliferative and pro-apoptotic effects have been indicated. KIF11 siRNA has been used as the positive control.

The mRNA expression of *AIM1*, *ERGIC1*, *TMED3*, and *TPX2* was studied in six prostate cancer (VCaP, PC-3, MDA-PCa-2b, LNCaP, DU145 and 22Rv1) and three non-malignant prostate epithelial cell lines (RWPE-1, PrEc, EP156T) (Figure 3A). Especially *ERGIC1* and *TMED3* were found to be highly expressed in the cancer but not in the non-malignant cell lines. Among the malignant cell lines *AIM1*, *ERGIC1*, and *TMED3* were most highly expressed in VCaP, and *TPX2* in LNCaP cells. Two siRNAs per gene, chosen based on the target silencing efficacy, were selected for validation studies (Figure 3B and Supporting Figure S2). The results from 72 h cell viability and apoptosis assay confirmed the antiproliferative effect of *TMED3* and *TPX2* silencing in both of the cell lines. As expected based on the screening results, *ERGIC1* had a role specifically in the ERG oncogene expressing VCaP cell viability. However, although AIM1 siRNAs were able to decrease VCaP cell viability, no consistent effects were observed in LNCaP cells (Figure 3C). The caspase 3/7 activity was enhanced mainly in response to *TPX2* and *TMED3* silencing in LNCaP cells, whereas *TPX2* and ERGIC1 silencing induced apoptosis in VCaP cells with both siRNAs (Figure 3D).

### *AIM1*, *ERGIC1*, and *TPX2* are Highly Expressed in Clinical Prostate Cancer Samples

**Figure 4:**
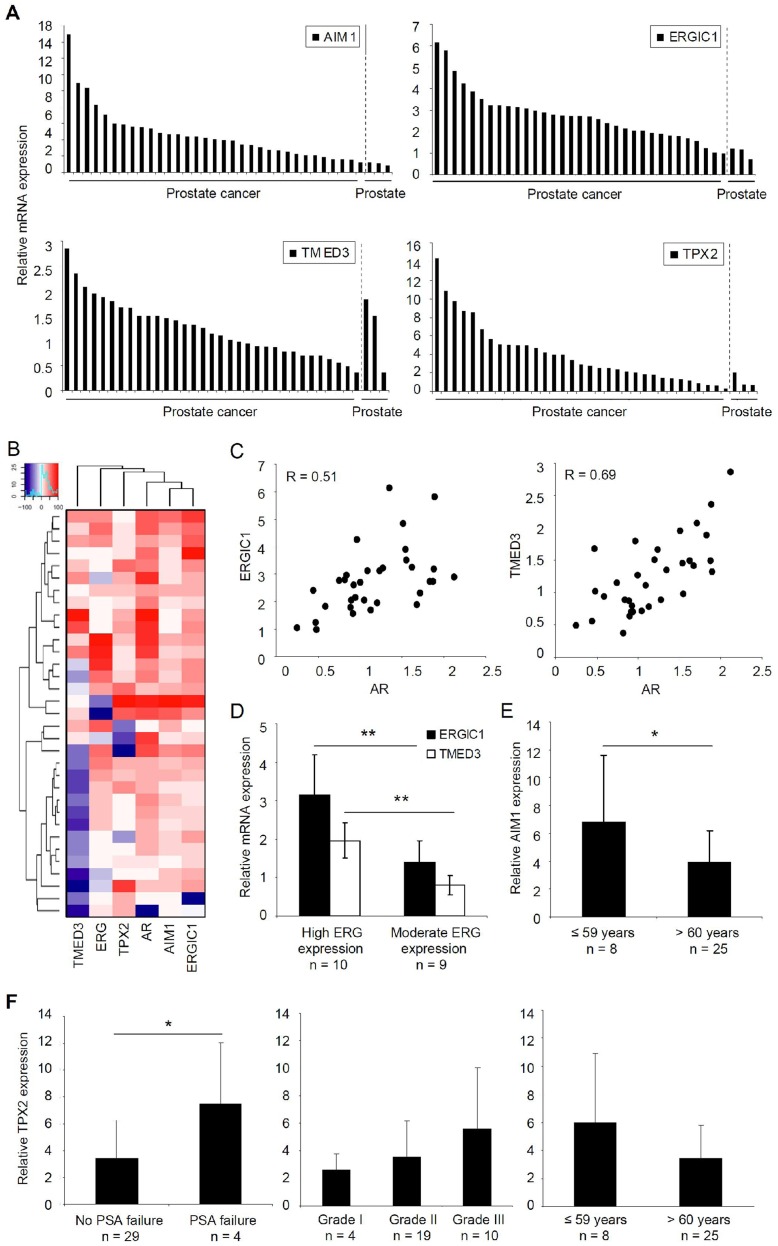
Validation of *AIM1*, *ERGIC1*, *TMED3* and *TPX2* expression in clinical prostate tissue samples. **A.** The mRNA expression of target genes in 33 primary prostate cancer and 3 non-malignant prostate tissue samples. The mean expression the non-malignant samples has been set as 1. **B.** Heatmap visualization of the gene-wise scaled relative mRNA expression values for *AIM1*, *ERGIC1*, *TMED3*, *TPX2*, *ERG*, and *AR* in 33 primary prostate cancer tissues. The heatmap is drawn based on unsupervised hierarchical clustering of the expression values. Relative mean expression level in normal control samples was set as 0. **C.** Co-expression patterns between ERGIC1 and AR mRNA, as well as TMED3 and AR mRNA in 33 primary prostate cancer samples. **D.** Association of ERGIC1 and TMED3 mRNA expression with ERG mRNA expression in the ERG positive primary prostate tumors (n = 19). **E.** Relative mRNA expression of *AIM1* in primary prostate cancer samples in comparison to patient age. **F.** Relative mRNA expression of *TPX2* in primary prostate cancer samples in comparison to occurrence of PSA failure, WHO tumor grade and patient age.

Validation of target gene expression patterns in clinical prostate samples confirmed that AIM1, ERGIC1, and TPX2 mRNA levels were significantly elevated in prostate cancer tissues (n = 33), compared to non-malignant control tissue samples (n = 3). All cancer samples expressed AIM1 mRNA at higher levels than any of the non-malignant samples; while ERGIC1 was over-expressed in 94% (n = 31), and TPX2 in 64% (n = 23) of the cancer samples. However, despite the promising results of TMED3 expression patterns in cultured prostate cells, TMED3 mRNA was expressed at equal levels in the non-malignant and cancer tissues (Figure 4A). For comparison, mRNA levels for the key prostate cancer oncogenes AR and ERG were also determined in the same clinical samples, and the results are presented as a heatmap in Figure 4B. Out of the four potential novel target genes, *ERGIC1* (R = 0.51) and *TMED3* (R = 0.69) expression patterns correlated most significantly with *AR* expression (Figure 4C). In addition, although ERGIC1 and TMED3 were highly expressed in both ERG negative and positive prostate cancers, their mRNA expression levels positively correlated with ERG expression levels in ERG positive samples (P = 0.002 and P = 0.007 respectively) (Figure 4D). Comparison of target gene expression with clinical parameters revealed that *AIM1* correlated significantly (P = 0.03) with young age (<60 years) (Figure 4E). In addition, high *TPX2* expression correlated with prostate-specific antigen (PSA) failure (P = 0.02), and associated with high WHO grade and young age (Figure 4F). No such associations were found with ERG1C1 or TMED3 mRNA expression.

### *AIM1*, *ERGIC1*, *TMED3*, and *TPX2* are all Regulated by *ERG* Oncogene and androgens in Cultured Prostate Cancer Cells

**Figure 5:**
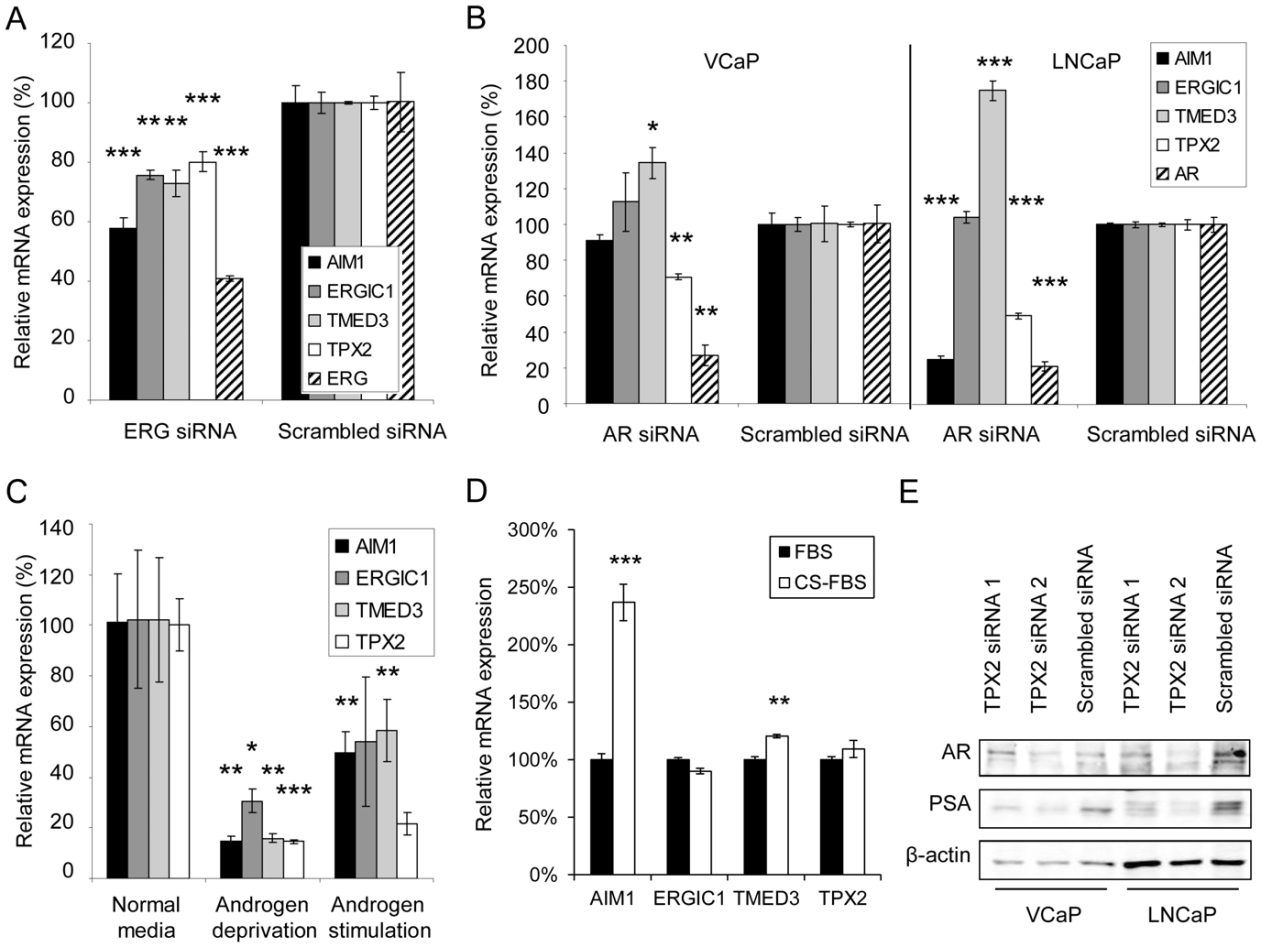
*AIM1*, *ERGIC1*, *TMED3* and *TPX2* expression interrelates with *ERG* and *AR* oncogene expression *in vitro*. **A.** The effect of 48 h *ERG* silencing on the expression of the target genes in VCaP cells. **B.** The effect of 48 h *AR* silencing on the expression of the target genes in VCaP and LNCaP cells. **C.** The effect of 24 h androgen deprivation and sequential 24 h androgen stimulation (10 nM R1881) on the expression the target genes in LNCaP cells. **D.** The level of target mRNA expression in LNCaP cells cultured in normal media (FBS) and in chargoal-stripped (CS-FBS) androgen ablated media. **E.** The effect of 72 h *TPX2* silencing on the protein expression of AR and PSA. β-actin has been used as a loading control. The statistical significance of the results in comparison to control experiment have been indicated.

To evaluate the potential role of ERG and AR in the regulation of these prostate cancer cell growth promoting genes, the effect of *ERG* and *AR* silencing, as well as androgen deprivation and stimulation on target gene expression was analyzed. Surprisingly, *ERG* silencing significantly decreased the mRNA expression of all four target genes in VCaP cells (Figure 5A). Furthermore, *AR* silencing decreased the mRNA expression of *AIM1* in LNCaP cells and *TPX2* in both VCaP and LNCaP cells, whereas the expression of TMED3 mRNA was increased (Figure 5B). Surprisingly, although *ERG1C1* expression was associated with *AR* and AR driven *ERG* expression in clinical prostate cancers, no major changes were observed in the expression of ERGIC1 mRNA expression in response to AR silencing. Despite the diverse effects of AR silencing on target gene expression, androgen deprivation decreased and the synthetic androgen R1881 induced the expression of all of the target genes in LNCaP cells in comparison to the expression levels detected in androgen deprived conditions (Figure 5C). The expression of the target genes was studied also in LNCaP derivatives cultured in stable androgen ablated conditions mimicking castration-resistant tumors. The results show a significant increase in AIM1 expression in the ablated cells in comparison to the parental cells cultured in normal media (Figure 5D).

Taken together, these results suggest that the expression of the potential novel drug targets *AIM1*, *ERGIC1*, *TMED3*, and *TPX2* is promoted by *ERG* oncogene and androgens in cultured prostate cancer cells. Furthermore, *AR* silencing decreases the expression of *AIM1* and *TPX2* in normal cell culture conditions, whereas *AIM1* expression is stimulated in cells cultured in androgen deprived conditions.

### TPX2 Inhibition Suppresses AR Signaling in Cultured Prostate Cancer Cells

Due to the function of *AR* as an important oncogene in prostate cancer, the effect of *AIM1*, *ERGIC1*, *TMED3*, and *TPX2* expression on AR signaling was analyzed. The results showed, that although no consistant changes were observed in the protein expression of AR and PSA in response to *AIM1*, *ERGIC1* and *TMED3* silencing, *TPX2* silencing was able to significantly reduce PSA expression in both VCaP and LNCaP cell lines, as well as to decrease AR expression in LNCaP cells (Figure 5E and Supporting Figure S3A). Furthermore, qRT-PCR results confirmed that *TPX2* regulates the expression of *AR* and *PSA* already at mRNA level (Supporting Figure S3B).

In order to illustrate the potential of the selected putative targets in the treatment of hormone-refractory disease, the efficacy of *AIM1*, *ERGIC1*, *TMED3*, and *TPX2* silencing in the inhibition of prostate cancer cells cultured in androgen deprived conditions was studied. The results support the potential of TPX2 in the treatment of castration-resistant tumors and highlight the induction of apoptosis due to AIM1 and TMED3 inhibition especially in the androgen independent cancer cells (Supporting Figure S4).

### ERGIC1 Silencing Reduces the mRNA Expression of ERG *in* *vitro*

Since *ERGIC1* and *TMED3* expression correlated with *ERG* expression levels in ERG positive primary prostate tumors, the potential effect of their expression on ERG mRNA expression was studied in VCaP cell line. The results indicated that *ERGIC1* silencing was able to systematically downregulate ERG mRNA expression, although the results did not reach statistical significance with both siRNAs studied (Supporting Figure S5).

### *In Silico* Co-expression Analysis Connects *AIM1*, *ERGIC1*, *TMED3* and *TPX2* to Carcinogenesis

To investigate the potential role of the four putative target genes in prostate cancer, *in silico* co-expression signatures in clinical prostate cancer samples were analysed (Table 1 and Supporting Table S3). The results showed that *ERGIC1* and *TMED3* are expressed in the same samples as genes involved in protein transport at ER and Golgi apparatus, whereas *TPX2* is expressed together with genes involved in mitosis. Furthermore, cancer was among the top disease processes associated with the co-expressed genes for both *ERGIC1* and *TPX2*. Genes co-expressed with *AIM1* locate in ribosomes and mitochondrion, and have a role in the regulation of cell morphology. In addition, high *AIM1 and TMED3* expression associates with genes involved in lipid metabolism, and high *ERGIC1 and TMED3* expression with genes involved in redox homeostasis.

**Table 1:** Functional gene ontology annotations for the genes co-expressed (R >0.5 and P<0.001) with *AIM1*, *ERGIC1*, *TMED3* or *TPX2* in clinical prostate cancer samples (n = 66–329).

Gene	Annotation	P-value
**AIM1**	**Cellular compartment**	
	Large ribosomal unit	9.0E-03
	Mitochondrion	4.2E-02
	**Disease**	
	Cardiovascular Disease	4,50E-04 - 3,48E-02
	**Molecular and cellular functions**	
	Cell Morphology	8,46E-04 - 4,72E-02
	Lipid Metabolism	2,89E-03 - 4,72E-02
	Molecular Transport	2,89E-03 - 4,72E-02
	**Biological Processes**	
	Transition metal ion transport	8.6E-04
	Protein oligomerization	9.8E-03
	Sterol metabolic process	2.1E-02
**ERGIC1**	**Cellular compartment**	
	ER-Golgi intermediate compartment	1.5E-05
	Mitochondrion	2.6E-05
	**Disease**	
	Cancer	1,55E-05 - 3,44E-02
	**Molecular and cellular functions**	
	Amino Acid Metabolism	5,46E-08 - 3,44E-02
	Small Molecule Biochemistry	5,46E-08 - 3,44E-02
	Energy Production	8,69E-06 - 3,44E-02
	**Biological Processes**	
	Carboxylic acid catabolic process	1.3E-09
	Oxidation reduction	1.3E-05
	Golgi vesicle transport	2.5E-05
**TMED3**	**Cellular compartment**	
	Endoplasmic reticulum	1.7E-08
	ER-Golgi intermediate compartment	1.5E-03
	**Disease**	
	Dermatological Diseases and Conditions	4,83E-03 - 4,71E-02
	**Molecular and cellular functions**	
	Lipid Metabolism	1,39E-03 - 4,72E-02
	Small Molecule Biochemistry	1,39E-03 - 4,72E-02
	Cell Morphology	2,32E-03 - 4,72E-02
	**Biological Processes**	
	Intracellular protein transport	2.3E-04
	Cell redox homeostasis	1.7E-04
	Regulation of caspase activity	5.2E-03
TPX2	**Cellular compartment**	
	Chromosome, centromeric region	1.1E-16
	Microtubule cytoskeleton	7.3E-15
	**Disease**	
	Cancer	2,88E-09 - 4,96E-02
	**Molecular and cellular functions**	
	Cell Cycle	1,01E-22 - 4,70E-02
	Cellular Assembly and Organization	1,11E-13 - 4,68E-02
	DNA Replication, Recombination, and Repair	1,11E-13 - 4,68E-02
	**Biological Processes**	
	M phase of mitotic cell cycle	9.4E-29
	Microtubule-based process	4.2E-11
	DNA metabolic process	8.8E-11

## Discussion

Accumulating gene expression data from human tissues provide important information for identification of novel biomarkers and drug targets for personalized medicine. In addition, high-throughput cell-based RNAi screening enables functional validation of the candidate drug targets in an efficient manner [24]–[26]. In this study, the potential of these techniques was combined in order to identify genes that play critical roles in regulating prostate cancer cell proliferation and viability. Moreover, the expression of the novel candidate drug targets was validated in a set of clinical prostate cancer samples to evaluate further their potential as targets for future personalized prostate cancer therapeutics.

A bioinformatic gene expression analysis was carried out using GeneSapiens database [27] to distinguish the most promising *in vivo* prevalidated prostate cancer drug targets for further studies in cultured prostate cancer cells. In total, 295 genes were selected based on their high mRNA expression levels in prostate, prostate cancer or in metastatic prostate cancer samples. By utilizing this gene expression based pre-selection approach instead of a commercial ready made siRNA libraries, we aimed at maximizing the focus on prostate and prostate cancer relevant genes. In addition, other possible benefits accomplished by pre-selecting the genes for RNAi functional assays include development of targeted, personalized and efficient therapies with less unwanted side-effects. RNAi based high-throughput functional profiling was performed using two prostate cancer cell lines. Since siRNAs are known to induce off-target effects [61], four siRNAs per gene were initially used. In addition, to validate the results, positive and negative controls were utilized, and the cell proliferation siRNA screen was conducted in triplicates in both of the cell lines. Furthermore, potential induction of apoptosis by the siRNAs was also evaluated to gain further confirmation, and the results from the functional assays were validated *in vitro* using two siRNAs per each target gene. As evidenced by the high rate of hit siRNAs especially in LNCaP cells, the focused approach was successful in maximizing the amount of potential prostate cancer relevant drug targets identified. In conclusion, the combinatorial usage of microarray and RNAi techniques yielded in a large number of putative novel drug targets, with biomarker potential, for future development of targeted and personalized prostate cancer management.

Based on RNAi screening results, genome-wide gene expression patterns and novelty *AIM1*, *ERGIC1* and *TMED3* and *TPX2* were selected for further validation. Validation experiments included target mRNA expression analysis in cultured prostate cell lines, as well as in clinical prostate samples. All of the four candidate targets were found to be highly expressed especially in the prostate cancer cell lines studied and showed highest expression either in VCaP or LNCaP cells, utilized in the HT RNAi screens. The clinical validation showed that the putative drug targets were widely expressed in clinical prostate cancer samples. Moreover, *AIM1*, *ERGIC1*, and *TPX2* were shown to be highly expressed specifically in prostate cancer tissues, thereby confirming the results of the bioinformatic surveys. Interestingly, even though *AIM1*, *ERGIC1*, *TMED3* and *TPX2* were partially expressed in separate subsets of prostate cancers, all of the candidate target genes were found to be regulated by *ERG* oncogene as well as androgens highlighting the significance of *ERG* and androgens in promoting prostate oncogenesis.

As reports of the role of *AIM1* in different cancers are controversial [43], [46]–[48], further studies are needed to evaluate its potential in cancer management. However, our results indicate that *AIM1* is highly expressed in primary prostate cancers as well as in cultured androgen-independent prostate cancer cells, and support the potential of AIM1 inhibition in prostate cancer management, most likely in combinatorial treatment approaches. Furthermore, the co-expression gene signature analysis supports the earlier report associating *AIM1* with the regulation of cell morphology and shape [42].

*ERGIC1* and *TMED3* expression associated with ER and Golgi apparatus function. Although inhibition of ER and Golgi function has been suggested a promising opportunity for targeted cancer therapy, *ERGIC1* and *TMED3* have not been previously described as candidate cancer targets [51], [52]. Moreover, this study associates *ERGIC1* and *TMED3* expression with *ERG* oncogene expression, supporting their potential in the management of prostate cancer. Since *ERGIC1* was highly expressed in most primary prostate tumors, and *ERGIC1* silencing was able to downregulate ERG expression, it is an intriguing potential drug target especially for the ERG oncogene expressing tumors. Previous study has shown that ETS (*ETS1*) transcription factor mediates adaptation to ER stress in melanoma cells [62], supporting the potential role of ERG in the regulation of ER function related genes in prostate cancer. Furthermore, the gene co-expression signatures indicate that *ERGIC1* and *TMED3* are expressed together with genes involved in cellular redox homeostasis, in agreement to our earlier results demonstrating that ERG oncogene expressing cancer cells are sensitive to oxidative stress inducers [30], [63]. Finally, both of the ER related genes were upregulated by androgens, supporting the earlier results suggesting, that the expression of ER stress response genes is regulated by androgen in prostate cancer cells [64].

*TPX2* has been proposed as a potential drug target in multiple cancer types [56]–[58], and our results reveal *TPX2* as a potent candidate drug target also in prostate cancer. We showed that *TPX2* is regulated by *AR* and androgens, and that *TPX2* silencing downregulates AR signaling. Furthermore, in accordance to the previous studies associating *TPX2* expression with poor survival in lung cancer and astrocytoma, as well as with aggressive disease in meningiomas [53]–[55], our results indicated that TPX2 expression associates with PSA failure, high tumor grade (WHO) and young age in prostate cancer. Taken together, TPX2 is a candidate therapeutic target in majority of prostate cancers, possibly also in advanced and castration-resistant disease.

In conclusion, this study illustrates the power of gene expression data analysis coupled with high-throughput RNAi in the exploration of potential novel target genes for cancer management. We present *ERGIC1* and *TMED3* as candidate drug targets for ERG oncogene positive tumors, whereas *TPX2* expression was associated with mitotic and aggressive disease. AIM1 was highly expressed in most of the prostate cancers studied, suggesting a broad therapeutic target group. Further studies are required to validate the *in vivo* therapeutic relevance of these promising targets. Furthermore, in addition to the four *in vitro* validated potential drug targets, the results from this study provide several other starting points for future preclinical and eventually clinical efforts to treat prostate cancer.

## Supporting Information

Figure S1 **The mRNA expression of** ***AIM1*** **,** ***ERGIC1*** **,** ***TMED3*** **and** ***TPX2*** **in clinical tissue samples based on the data available in GeneSapiens database.**
(PDF)

Figure S2 **Validation of target gene silencing of ERGIC1, TMED3 and TPX2 at protein level.** β-actin has been used as a loading control.
(PDF)

Figure S3 **A.** The effect of *AIM1*, *ERGIC1* and *TMED3* silencing on the protein expression of AR and PSA in VCaP and LNCaP cells. β-actin has been used as a loading control. **B.** The effect of TPX2 silencing on the mRNA expression of AR and PSA in LNCaP cells.
(PDF)

Figure S4 **The effect of 72 h target gene silencing on cell viability and induction of apoptosis in LNCaP derivatives cultured in normal serum containing media (FBS) and in androgen ablated media (CS-FBS).** The results have been compared to scrambled siRNA induced changes and the significance of the anti-proliferative and pro-apoptotic effects have been indicated. KIF11 siRNA has been used as the positive control.
(PDF)

Figure S5 **The effect of** ***ERGIC1*** **silencing on the mRNA expression of** ***ERG*** **.**
(PDF)

Table S1 **Primers and probes utilized in qRT-PCR analysis.**
(PDF)

Table S2 **The results from the siRNA cell viability and apoptosis assays in VCaP and LNCaP cell lines.** The results are presented as B-score, and the results exceeding the hit limit (-2 SD in cell viability and 3 SD in apoptosis) have been indicated with colour.
(XLS)

Table S3 **The genes co-expressed (R >0.5 and P<0.001) with** ***AIM1*** **,** ***ERGIC1*** **,** ***TMED3*** **or** ***TPX2*** **in clinical prostate cancer samples (n = 66–329)** ***in silico*** **, and utilized in** **Table 1** **.**
(XLS)
