# Supplementary figures and images for: High-Throughput Transcriptomic and RNAi Analysis Identifies AIM1, ERGIC1, TMED3 and TPX2 as Potential Drug Targets in Prostate Cancer
Source: PLoS One. 2012 Jun 28;7(6):e39801. doi: 10.1371/journal.pone.0039801 (PMC3386189; doi:10.1371/journal.pone.0039801)

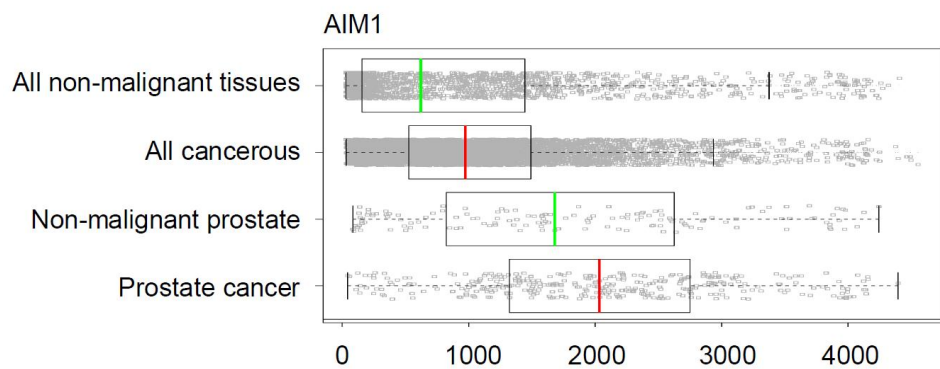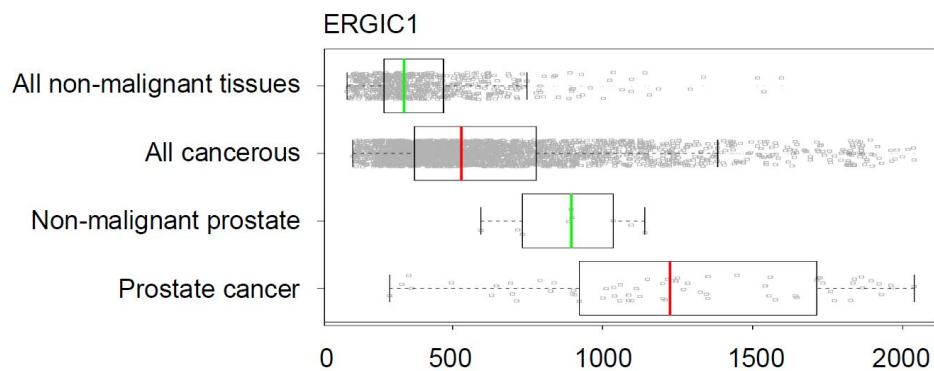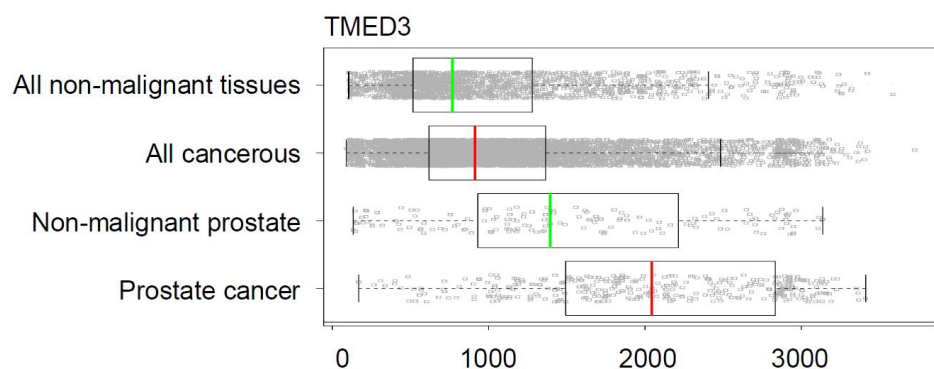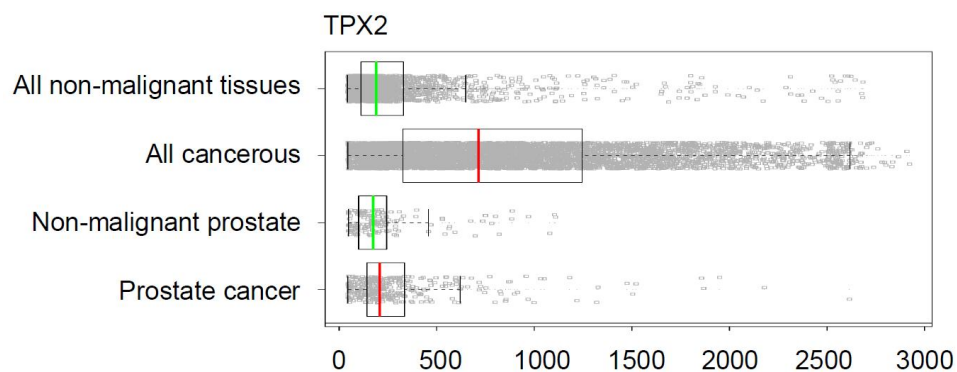

Supplement: Figure S1 — The mRNA expression of AIM1 , ERGIC1 , TMED3 and TPX2 in clinical tissue samples based on the data available in GeneSapiens database. (PDF) [file pone.0039801.s001.pdf]

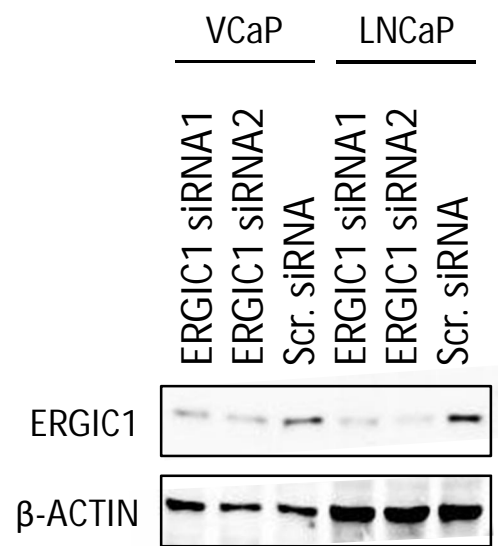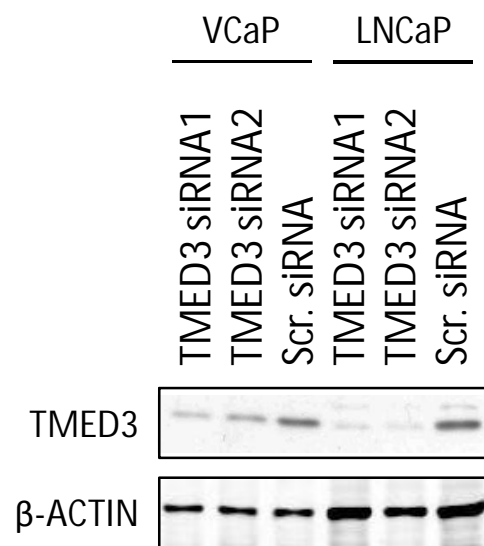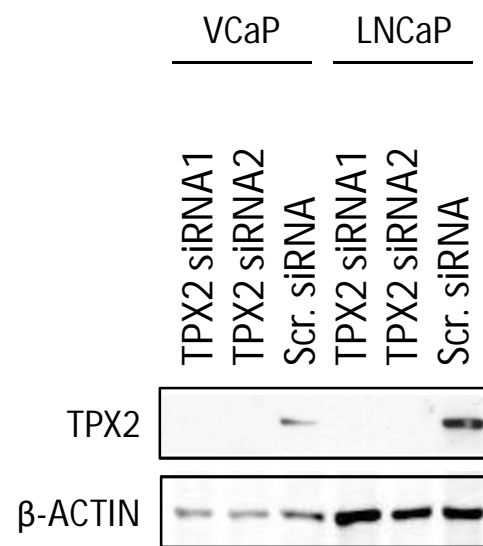

Supplement: Figure S2 — Validation of target gene silencing of ERGIC1, TMED3 and TPX2 at protein level. β-actin has been used as a loading control. (PDF) [file pone.0039801.s002.pdf]

A

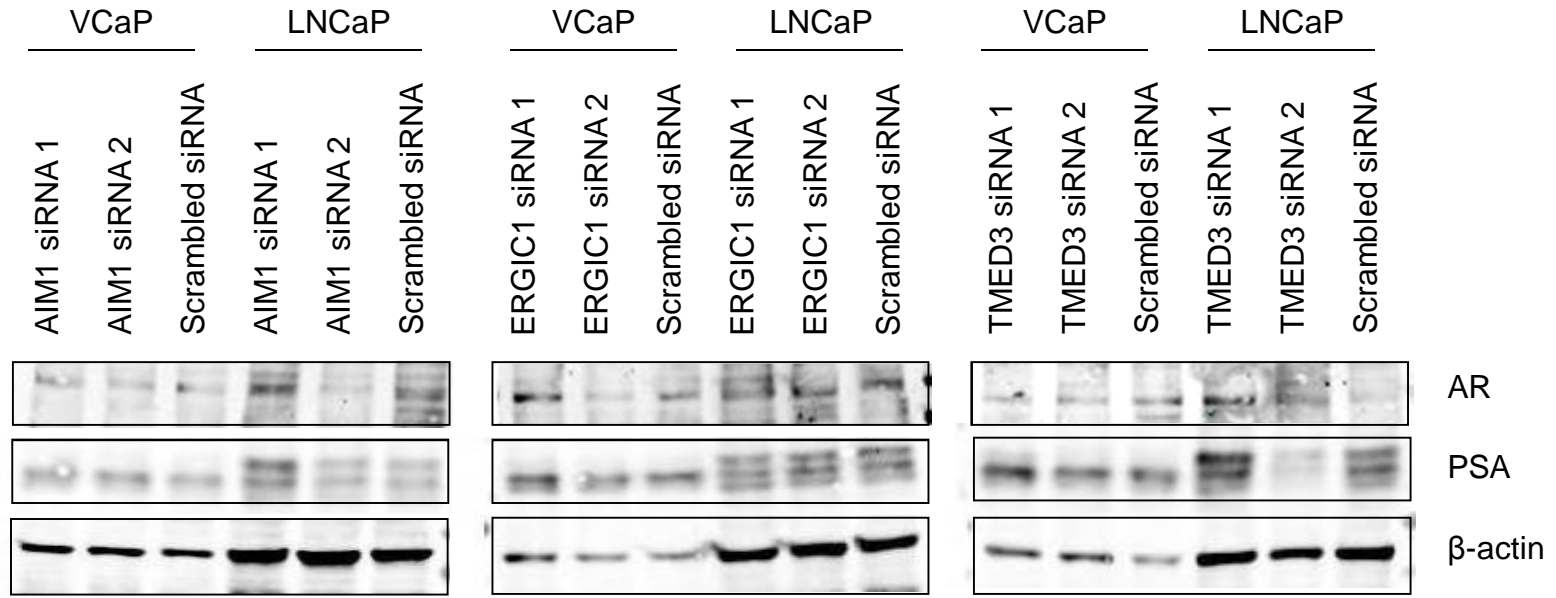

B

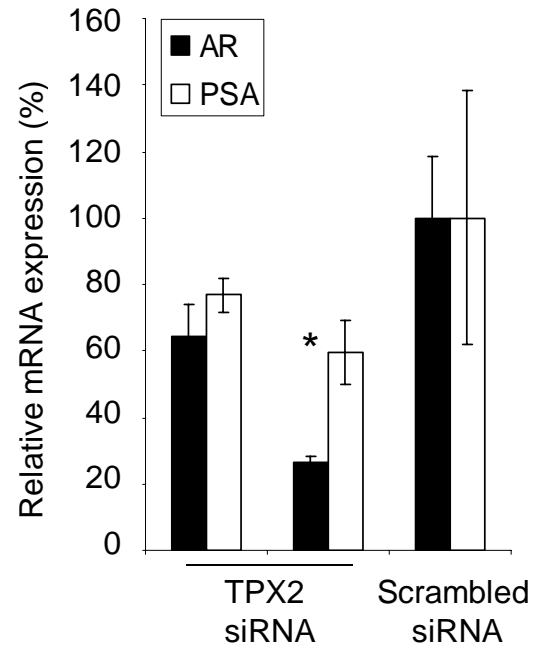

Supplement: Figure S3 — A. The effect of AIM1, ERGIC1 and TMED3 silencing on the protein expression of AR and PSA in VCaP and LNCaP cells. β-actin has been used as a loading control. B. The effect of TPX2 silencing on the mRNA expression of AR and PSA in LNCaP cells. (PDF) [file pone.0039801.s003.pdf]

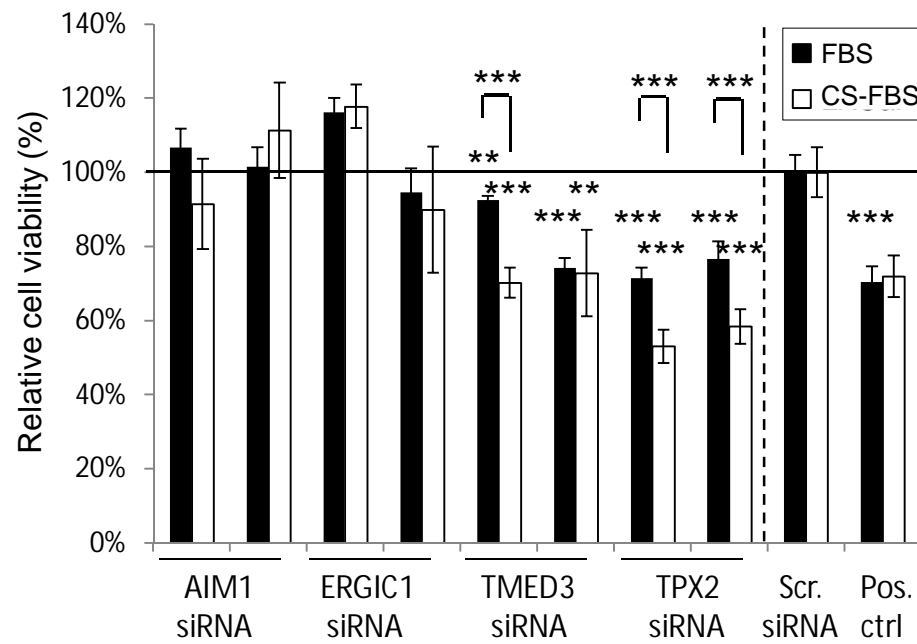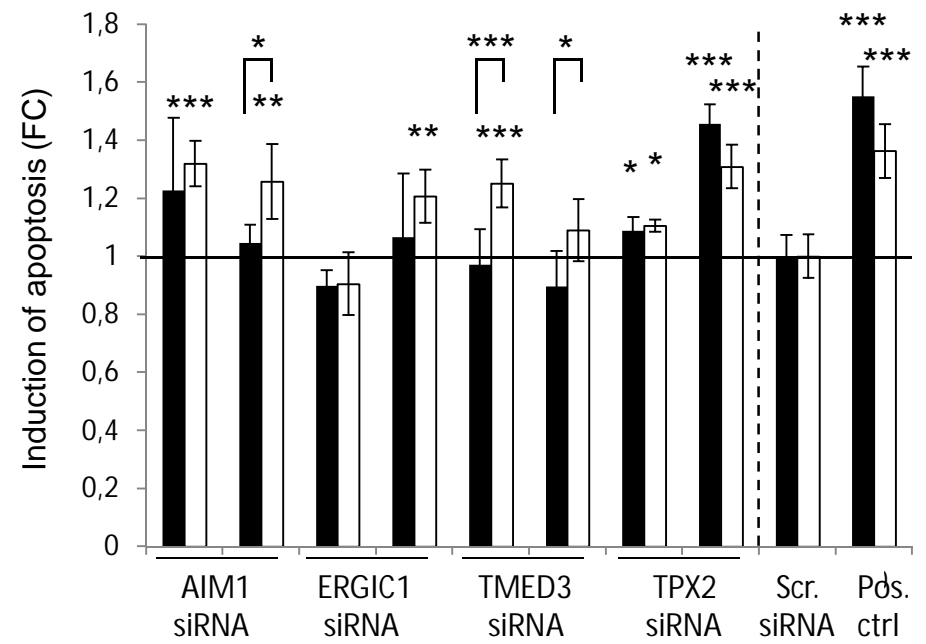

Supplement: Figure S4 — The effect of 72 h target gene silencing on cell viability and induction of apoptosis in LNCaP derivatives cultured in normal serum containing media (FBS) and in androgen ablated media (CS-FBS). The results have been compared to scrambled siRNA induced changes and the significance of the anti-proliferative and pro-apoptotic effects have been indicated. KIF11 siRNA has been used as the positive control. (PDF) [file pone.0039801.s004.pdf]

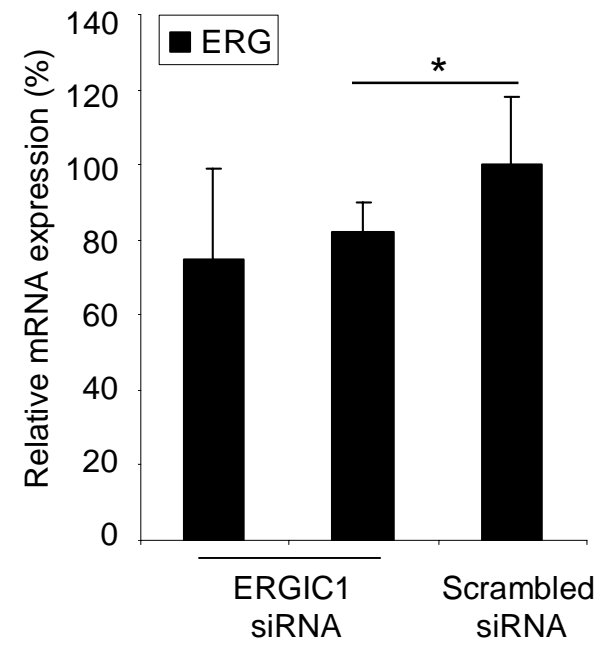

Supplement: Figure S5 — The effect of ERGIC1 silencing on the mRNA expression of ERG . (PDF) [file pone.0039801.s005.pdf]
