# Supplementary material for: High-Throughput Transcriptomic and RNAi Analysis Identifies AIM1, ERGIC1, TMED3 and TPX2 as Potential Drug Targets in Prostate Cancer
Source: PLoS One. 2012 Jun 28;7(6):e39801. doi: 10.1371/journal.pone.0039801 (PMC3386189; doi:10.1371/journal.pone.0039801)
Supplement: Table S1 — Primers and probes utilized in qRT-PCR analysis. (PDF) [file pone.0039801.s006.pdf]

Supporting Table S1. Primers and probes utilized in qRT-PCR analysis.

Gene	Forward	Reverse	Probe
ACTB	ccaaccgcgagaagatga	ccagaggcgtacagggatag	64
AIM1	ctggaatgtcattatcagacacaa	tcagagacgtcgggttcact	85
AR	gccttgctctctagcctcaa	gtcgtccacgtgtaagttgc	14
ERG	caggtgaatggctcaagga	agttcatcccaacggtgtct	44
ERGIC1	agtacacggtggccaacaa	aaccagattgcagggatgat	5
TMED3	gggttctctgtacctgaggaaa	caccgagggtgagcagat	81
TPX2	acatctgaactacgaaagcatcc	ggcttaacaatggtacatccctta	51
